# Global Reach of Direct-to-Consumer Advertising Using Social Media for Illicit Online Drug Sales

**DOI:** 10.2196/jmir.2610

**Published:** 2013-05-29

**Authors:** Tim Ken Mackey, Bryan A Liang

**Affiliations:** ^1^Institute of Health Law StudiesCalifornia Western School of LawSan Diego, CAUnited States; ^2^University of California, San Diego-San Diego State UniversityJoint Doctoral Program in Global HealthLa Jolla, CAUnited States; ^3^San Diego Center for Patient SafetyDepartment of AnesthesiologyUniversity of California, San DiegoSan Diego, CAUnited States

**Keywords:** health policy, pharmacies, social media, Internet, social marketing, marketing of health services, online pharmaceutical services

## Abstract

**Background:**

Illicit or rogue Internet pharmacies are a recognized global public health threat that have been identified as utilizing various forms of online marketing and promotion, including social media.

**Objective:**

To assess the accessibility of creating illicit no prescription direct-to-consumer advertising (DTCA) online pharmacy social media marketing (eDTCA2.0) and evaluate its potential global reach.

**Methods:**

We identified the top 4 social media platforms allowing eDTCA2.0. After determining applicable platforms (ie, Facebook, Twitter, Google+, and MySpace), we created a fictitious advertisement advertising no prescription drugs online and posted it to the identified social media platforms. Each advertisement linked to a unique website URL that consisted of a site error page. Employing Web search analytics, we tracked the number of users visiting these sites and their location. We used commercially available Internet tools and services, including website hosting, domain registration, and website analytic services.

**Results:**

Illicit online pharmacy social media content for Facebook, Twitter, and MySpace remained accessible despite highly questionable and potentially illegal content. Fictitious advertisements promoting illicit sale of drugs generated aggregate unique user traffic of 2795 visits over a 10-month period. Further, traffic to our websites originated from a number of countries, including high-income and middle-income countries, and emerging markets.

**Conclusions:**

Our results indicate there are few barriers to entry for social media–based illicit online drug marketing. Further, illicit eDTCA2.0 has globalized outside US borders to other countries through unregulated Internet marketing.

## Introduction

An estimated 2.4 billion persons worldwide used the Internet in 2011 [1,2]. This increasing use of the Internet and its related technologies has enabled the rise of social media platforms, including Facebook, Twitter, MySpace, and Google+. Collectively, these types of interactive systems are also known as *Web 2.0*. The phenomenon is global, with close to 4 of 5 active worldwide Internet users regularly visiting social media, and social media platforms representing the top online destinations in a cross-section of 10 developed countries and emerging markets (including Brazil, Mexico, India, and China) [1,3,4].

The increased use of the Internet and social media technology has also been associated with direct-to-consumer advertising (DTCA) to market health-related products [5,6]. From 1996 to 2005, DTCA pharmaceutical expenditures in the United States experienced rapid increases estimated at 330% with a total of US $4 billion in spending for 2009 [7,8]. However, recent market surveys indicate that American DTCA expenditures have recently experienced modest declines largely because of the global economic recession and patent expiration of blockbuster drugs [1,2]. Despite the overall declines in DTCA expenditures, Internet-based DTCA (eDTCA) has experienced increases in investment and attention, indicating a transition in marketing strategies from traditional media (eg, TV, radio, and print) to digital media [1,3,4].

Importantly, DTCA is generally banned in all developed countries, with the exception of the United States and New Zealand [5,6]. In the US, the Food and Drug Administration (FDA) has made efforts to provide industry guidance regarding off-label promotion over the Internet, but has yet to issue explicit guidance on regulation of other forms of Internet advertising, specifically social media-based promotion [7,8]. This lack of adequate DTCA regulation in the largest country where it is permitted may be leading to global dissemination through digital platforms that are not restricted by geopolitical borders [5,6]. This growing eDTCA use and limited FDA online advertising guidance also coincides with the increasing use of the Internet for sourcing of health information. A Pew Internet survey indicates 72% of US users search for health and medical information online, and approximately one-third of users attempt to use the Internet to self-diagnose their health issues [9].

The increased access and use of unregulated eDTCA by illicit actors, specifically online drug sellers, is even more disturbing. This includes social media-based eDTCA (eDTCA2.0) to market a wide variety of medical products that are of questionable quality, origin, and authenticity [5,6,10-12]. The use of eDTCA by these actors is problematic because it often contains content that is misleading, fraudulent, and otherwise illegal [5,13]. This includes the marketing and sale of prescription drugs as “no prescription necessary,” use of unsubstantiated medical questionnaires in lieu of a prescription, and marketing products as generic even if there is not an equivalent generic formulation [5,13,14].

Importantly, illicit online pharmacy activities and lack of appropriate eDTCA marketing regulation is compounded by an absence of online pharmacy regulation globally [14]. A recent World Health Organization (WHO) survey of member states found that 66% of respondents failed to specifically regulate Internet pharmacy operations [14]. This is further exacerbated by results from a recent FDA survey indicating that 23% of Internet consumers reported purchasing prescription drugs online [15]. In addition, there are globally established public health risks associated with illicit online sellers that have recently been further highlighted by national drug regulators, international organizations such as the WHO, the United Nations Office of Drugs and Crime, the International Criminal Police Organization, and other diverse stakeholders [16,17]. Hence, although there has been increasing global attention, the combination of unregulated online pharmacies and their use of digital marketing poses unique public health and patient safety risks, the scope and reach of which have yet to be adequately assessed.

Consequently, we wished to explore the potential ease of creation and reach of global, illicit online pharmacy eDTCA. Specifically, we differentiate illicit online drug sellers from licit sellers by defining illicit as those marketing and offering for sale prescription pharmaceutical products without the need for a prescription. Since the vast majority of countries require regulated pharmaceutical products be dispensed with a valid prescription from an authorized health professional, purported “no prescription” online sales are illegal and illicit activity. We also note pharmaceutical products dispensed with a valid prescription by an unlicensed or otherwise unauthorized provider may also constitute a violation of applicable laws, rules, and regulations. Further, because of the potential global reach of social media marketing in enabling this illicit trade, we also wished to assess the financial cost to develop illicit no prescription eDTCA2.0 marketing in these popular platforms and its potential global reach using commercially available Web tools and services. Finally, we were interested in determining whether our social media study sites would be taken down or blocked by service providers.

## Methods

Our overall strategy was to create an illicit online pharmacy social media presence in identified leading social media platforms. The primary outcomes of the study were to determine the up-front financial cost, determine if we could generate Internet/user traffic, and if so, its volume and geographic distribution.

**Figure 1:**
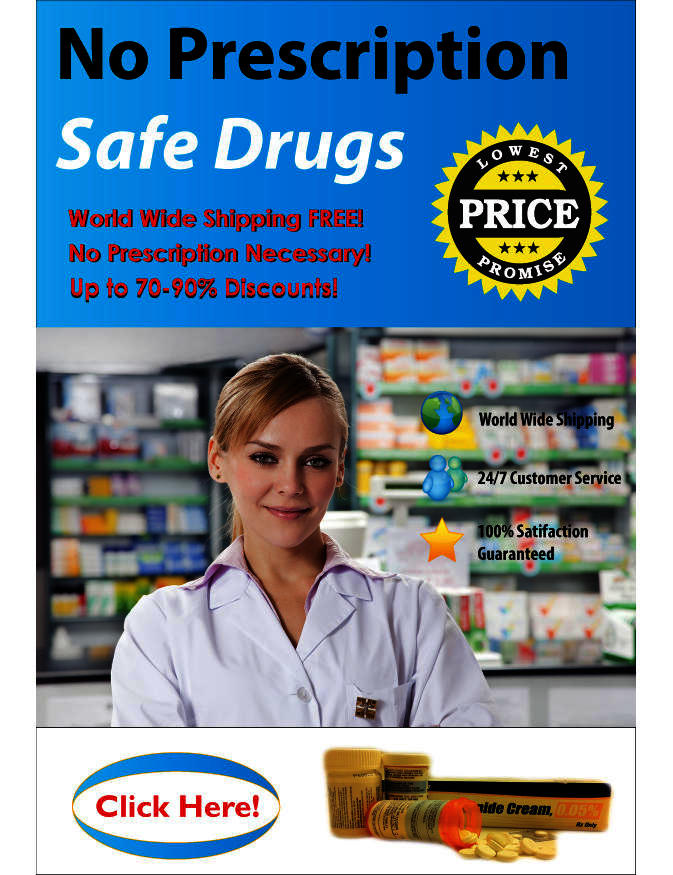
Illicit Internet-based direct-to-consumer advertising (eDTCA) ad created for study.

We first identified the top 4 social media platforms by traffic volume allowing user-generated eDTCA2.0 content at no cost or charge (ie, site tools that did not require sponsored or ad-based social media promotion; eDTCA sites) [18,19]. For each of these platforms, we created a fictitious advertisement marketing the sale of no prescription pharmaceuticals online (illicit eDTCA ad; see Figure 1). The advertisement was created with Adobe Photoshop CS4 software (San Jose, CA, USA). Content was consistent with prior studies identifying consumer-targeted messaging used by illicit online drug sellers, including the keywords no prescription, no RX, discounts, 100% satisfaction guaranteed, and other forms of potentially illegal, fraudulent, and misleading forms of marketing [20,21]. Images, icons, and a fictitious “lowest price” seal were also used, consistent with other forms of misleading promotion that we have observed being employed by suspect online drug sellers [5,10,16]. A stock image of a health professional was purchased for the advertisement to substantiate simulated commercial marketing.

To operationalize our online social media presence to measure user traffic and distribution, we purchased unique URLs with descriptive terms associated with illicit online drug sales and website space that terminated on these URLs (Table 1). Each unique URL corresponded to the specific social media advertisement, so that all visits to a particular Web address were linked to 1 social media platform point of advertisement. When clicked, rather than linking to an actual illicit online drug seller, all advertisements linked to static text content residing on the URL indicating “site unavailable.”

**Table 1:** Unique URLs linked to illicit Internet-based direct-to-consumer advertising (eDTCA) sites.

Study site URLs^a^	eDTCA platform and associated link
www.norxsafedrugsnow.com	Facebook (http://www.facebook.com/pages/No-Prescription-Online-Pharmacy/178946562182447)
www.norxneededsafedrugs.com	Twitter (@NoRXPharmacy)
www.norxneededcheapandsafedrugs.com	MySpace (http://www.myspace.com/norxonlinepharmacy)
www.norxneededcheapdrugs.com	Google+ (used with Gmail account: norxonlinepharmacy@gmail.com which was suspended)

^a^URLs hosted using Go Daddy services [22].

To assess geographic distribution, we also purchased commercially available site analytic services to track the number and location of visitors to each site. All these services (eg, URLs/domain names, website hosting, and site analytic services) were purchased from the popular Internet service and hosting site Go Daddy [22] given its convenience, market-leading presence, and large-scale commercial and personal usage. Go Daddy administers over 55 million domain names, has 11 million customers, and is the largest Internet Corporation for Assigned Names and Numbers (ICANN) domain registrar worldwide [22,23].

Using these illicit eDTCA ads with clear characteristics of questionable authenticity and legality, we then created user accounts on eDTCA sites using a fictitious email address. Sites were created using the descriptive term “NoRX Online Pharmacy” in site user registration fields. In addition, we registered our eDTCA Facebook site as a Brand or Product page under the category “Drugs.” These registration categories indicate Facebook has systems that specifically enable promotion of health/pharmaceutical-related products.

**Figure 2:**
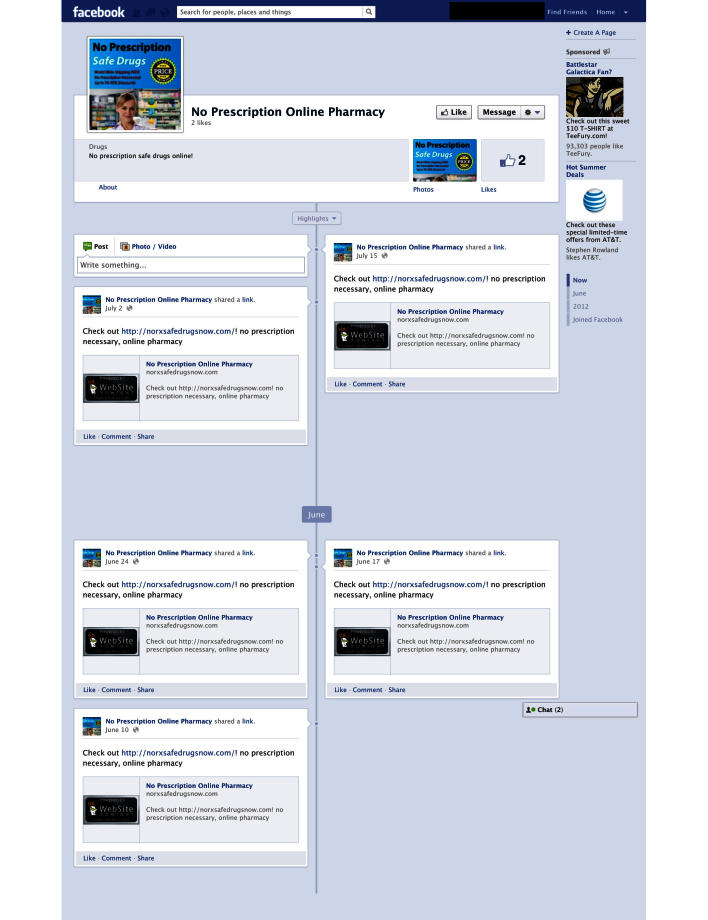
Facebook eDTCA site.

**Figure 3:**
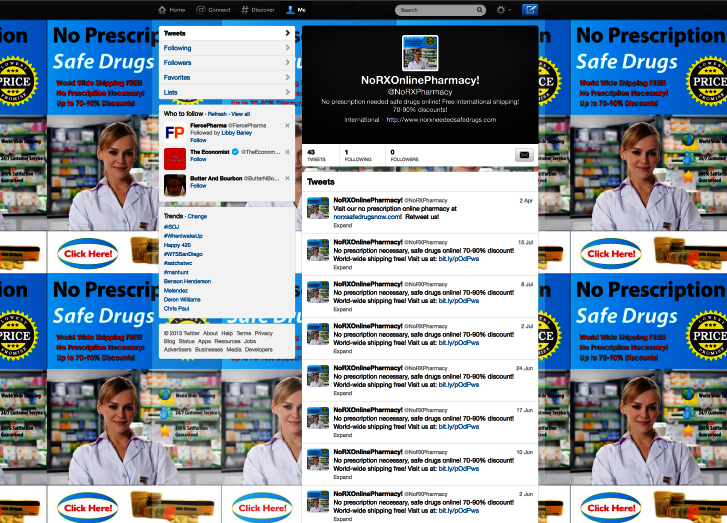
Twitter eDTCA site.

**Figure 4:**
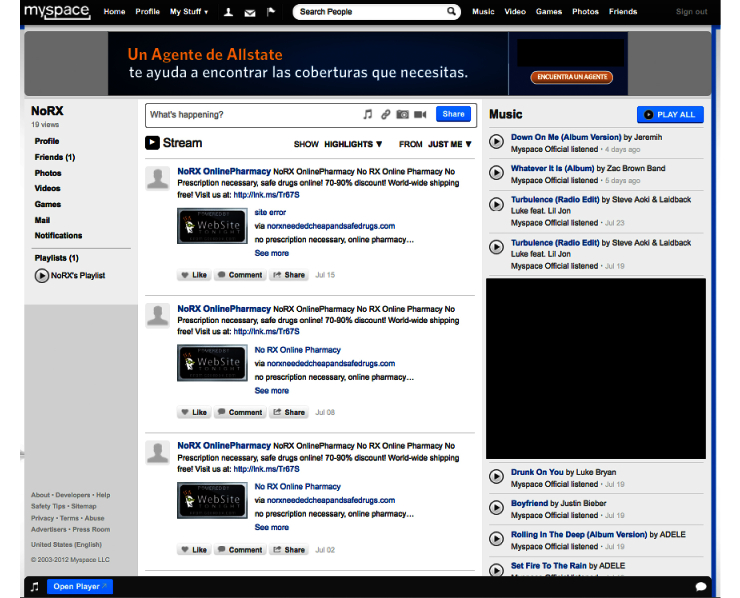
MySpace eDTCA site.

We then posted the illicit eDTCA ad to each eDTCA site and associated it with a link to the unique URLs/websites hosted by the Go Daddy services (Figures 2-4). On a weekly basis, we posted updates/messages advertising a no prescription online pharmacy and provided a link to the corresponding unique URLs/websites. Site analytics were reviewed on a biweekly basis with results aggregated into monthly traffic and geographic statistics. Employing the website analytic services, we tracked the 2 main study outcome measures: user traffic visiting illicit eDTCA ad-linked URLs, and the location of source Internet protocol (IP) addresses. User traffic is divided into 2 categories: the total number of visits to the site (including returning visitors), and unique user traffic, ie, the number of unique visitors to the site (excluding returning visitors). These definitions are used by site analytics tools and are consistent with industry standards.

Illicit eDTCA ads and websites went live on September 24, 2011, and data were collected for the period beginning September 24, 2011 and ending July 24, 2012. Data were presented by Go Daddy analytics anonymously, and no user or user-identifiable information was collected. Informed consent was exempted pursuant to US federal regulations 45 CFR § 46.101(b)(4), (c)(2), and (d), and this study protocol was approved by the California Western School of Law Institutional Review Board.

## Results

We identified the top 4 social media platforms enabling eDTCA2.0: Facebook, Twitter, MySpace, and Google+. Financial costs for creating illicit eDTCA ads across these platforms were relatively minor. In total, our outlays were less than US $400, with a cost of approximately US $80/eDTCA site (excluding the cost of existing software used to create the illicit eDTCA ad), inclusive of the fees for domain registration, website hosting and design tools, and website analytics (1-year service term), plus approximately US $60 for a limited commercial license for use of the health professional image used in the eDTCA2.0 ads.

We found that there appears to be little regulation of eDTCA2.0 content by social media platforms. Facebook, Twitter, and MySpace content created for the purposes of this study continue to remain accessible as of April 19, 2013, despite highly questionable and likely illegal content. Terms and conditions of use of these social media sites may also have been violated during the study, although no enforcement resulted (see Multimedia Appendix 1). Only our Google+ site was suspended by the service provider for undisclosed reasons. When trying to log into our Google+ account, we received a notification that our account had been disabled on October 26, 2011 (approximately 4 weeks after the site became active), at which point we terminated data collection. However, no reason was given for disabling our account. Others have had clearly legal Google+ accounts disabled during our study period for no apparent reason [24].

Fictitious illicit eDTCA ads linked to the remaining eDTCA sites (Facebook, Twitter, and MySpace) marketing illegal sale of prescription drugs generated total user traffic in the aggregate of 4107 visits, and unique user traffic in the aggregate of 2795 visits over a 10-month period from September 24, 2011 to July 24, 2011 for all websites. Select descriptive statistics on Web traffic over the study period had a mean monthly user traffic volume of 111 to 141 visits (upper and lower limits) and 78 to 97 visits for unique user traffic volume (Table 2).

**Figure 5:**
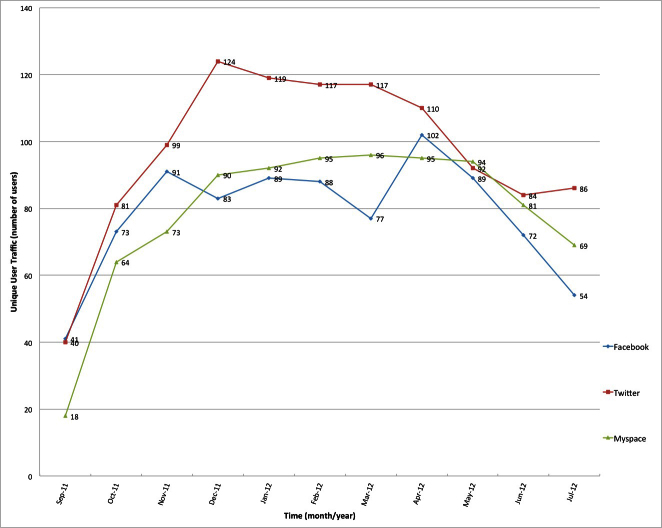
Unique user traffic statistics for eDTCA sites (provided by Go Daddy website analytics tool).

Our unique URLs/websites linked to illicit eDTCA ads posted on our Twitter eDTCA site generated the most user and unique user traffic volume of all platforms over the study period (Figure 5).

**Figure 6:**
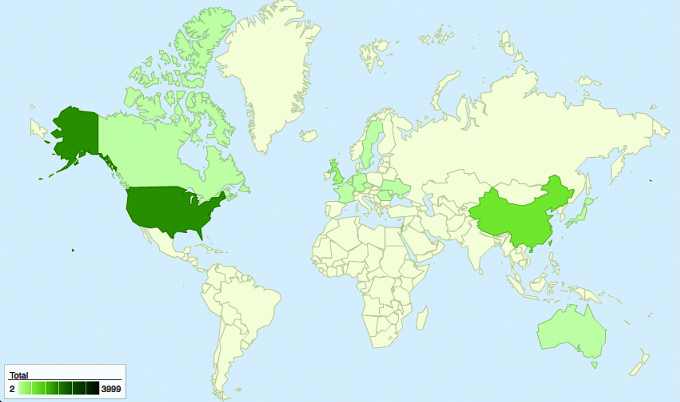
Geographic heat map of user traffic (source: Google Fusion Tables).

Country-level location data of user visits to the unique URLs/websites linked to illicit eDTCA ads resulted in user traffic from a total of 18 unique countries, including high-income, upper-middle income, lower-middle income, and emerging markets (Figure 6). The United States generated the highest percentage of visitors (54.0% of total traffic). Emerging markets of China and the Russian Federation ranked second (26.0%) and fourth (6.5%), respectively, with another high-income market country, the United Kingdom, ranking third (8.9%) in total visitors. Collectively, these top 4 countries generated the vast majority of user traffic (95.6%). Certain country visitors were unique to types of social media platforms, including Sweden (Facebook) and South Korea (Twitter; see Table 3).

**Table 2:** Illicit eDTCA unique user descriptive statistics.

Social media	Visits (n)	Mean	Median	Mode	Min	Max	Other information
Facebook	859	78.09	83	89	41	102	2 likes
Twitter	1069	97.18	99	117	40	124	1 following
MySpace	867	78.81	90	95	18	96	N/A

**Table 3:** Illicit eDTCA user location data.

Country	Classification^a^	Social media site, n (%)			
		Facebook	Twitter	MySpace	Total
United States	High income	702 (34.2)	818 (37.7)	650 (30.0)	2170 (54.3)
China	Upper-middle income/emerging market	240 (23.1)	412 (39.7)	387 (37.2)	1039 (26.0)
United Kingdom	High income	105 (8.8)	145 (9.6)	106 (8.2)	356 (8.9)
Russian Federation	Upper-middle income/emerging market	67 (5.6)	76 (5.0)	117 (9.0)	260 (6.5)
Unknown	—	38 (3.2)	12 (0.8)	7 (0.5)	57 (1.4)
Germany	High income	7 (0.6)	9 (0.6)	8 (0.6)	24 (0.6)
Japan	High income	5 (0.4)	15 (1.0)	4 (0.3)	24 (0.6)
Netherlands	High income	4 (0.3)	5 (0.3)	5 (0.4)	14 (0.4)
France	High income	4 (0.3)	4 (0.3)	4 (0.3)	12 (0.3)
Ukraine	Lower-middle income/emerging market	2 (0.2)	2 (0.1)	2 (0.2)	6 (0.2)
Israel	High income	2 (0.2)	2 (0.1)	2 (0.2)	6 (0.2)
Czech Republic	High income	2 (0.2)	2 (0.1)	2 (0.2)	6 (0.2)
Moldova, Republic of	Lower-middle income	3 (0.3)	2 (0.1)	2 (0.2)	7 (0.2)
Canada	High income	3 (0.3)	1 (0.1)	1 (0.1)	5 (0.1)
Sweden	High income	2 (0.2)	0 (0.0)	0 (0.0)	2 (0.1)
Australia	High income	1 (0.1)	1 (0.1)	1 (0.1)	3 (0.1)
Taiwan	High income^b^	1 (0.1)	1 (0.1)	1 (0.1)	3 (0.1)
Romania	Upper-middle income	1 (0.1)	1 (0.1)	1 (0.1)	3 (0.1)
Korea, Republic of	High income	0 (0.0)	2 (0.1)	0 (0.0)	2 (0.1)

^a^Country income classification based on World Bank List of Economies [25]. Country emerging market classification based on the International Monetary Fund’s World Economic Outlook Update [26].

^b^Taiwan is not listed as a separate country, but it is separately classified by the World Bank as a high-income country [27].

## Discussion

### Implications

It is inexpensive and easy to create an illicit online pharmacy marketing presence using eDTCA2.0. Moreover, there appears to be few barriers of entry and continued market presence of illegally marketed no prescription drugs using eDTCA2.0 on Facebook, Twitter, and MySpace.

These results are consistent with previous studies identifying an increasing presence of eDTCA2.0 use by illicit online drug sellers for a variety of medical products [5,6,10-12,28], but expand earlier work by finding consistent cross-platform presence in key social media platforms. This ease of creating a potential pharmaceutical criminal presence on globally accessible social media is highly alarming given its increasing utilization by online users and current lack of effective regulation [29]. Importantly, we believe these results are the first to demonstrate the use of social media platforms and Web tools to inexpensively create illicit eDTCA2.0 marketing and sites that are readily available and accessible to a worldwide audience.

Supporting study findings of ease of accessibility and affordability, social media eDTCA site registration was available at no cost, and illicit eDTCA ads creation and website maintenance were accomplished at a nominal cost for domain registration, website hosting and design tools, and website analytics—all conveniently packaged by the same provider. Access to social media allowed us to generate user traffic without employing forms of search engine optimization and search engine marketing services, avoiding these additional costs.

Our illicit eDTCA ads and websites generated relatively limited traffic compared with other traditional sources of e-commerce, such as legitimate pharmacy websites. This was likely due to a lack of site optimization and investment in Internet marketing, and our nonfunctional sites—we did not actually illicitly sell drugs online. Further, illustrating the ubiquity of illicit eDTCA2.0 presence, when registering our eDTCA sites, we discovered numerous other illicit online drug sellers utilizing social media platforms that competed directly with our study sites The presence of these more established illicit sites, and their aggressive forms of eDTCA marketing (ie, use of more explicit images/claims), further limited visibility and user traffic to our sites. However, despite these challenges, our illicit eDTCA ads and eDTCA sites were still able to generate traffic from global users.

Beyond safety concerns from consumer purchase of medicines from illicit online drug sellers, global dissemination of our eDTCA ads potentially violates DTCA prohibitions in virtually all countries other than the United States and New Zealand. Hence, global dissemination of eDTCA appears unrestricted and lacks necessary effective regulation and enforcement. Study results indicate our illicit eDTCA ads, which were clearly false and misleading, were accessed by users in diverse countries including high-income and middle-income countries, and other emerging markets.

We also observed user traffic that was highly concentrated in countries known to have illicit online drug sales and counterfeit drug trafficking activity, including China and Russia, and high-income consumption markets of the United States and the United Kingdom, where counterfeit seizures and incidents have been reported [13,16]. As well, the United States had the highest user traffic, which is also consistent with data supporting a high-proportion of US-based Internet and social media users and high prescription drug expenditures [29,30]. For further information on geographical findings, see Multimedia Appendix 1.

Although previous studies have reported findings supporting the negative impact of transborder promotion of traditional forms of DTCA (eg, satellite TV broadcasts and print content) [31-34], our study provides evidence of the globalization of illicit, broad-based digital forms of social media marketing. The results from this study can provide important information to global policymakers about necessary elements for appropriate illicit eDTCA regulation. For example, the use of high-risk keywords and terms in usernames and eDTCA2.0 content makes one direct solution clear: requiring social media service providers to monitor for content associated with illicit online drug selling activity during registration and in content generated by users via their sites, tools, and applications. We note that during the course of the study registration, use, and promotion, we consistently used terms that could easily identify the eDTCA site as being involved in potentially illegal activity (eg, “no prescription” and “no RX”). Social media providers should actively monitor for these keywords and proactively shut down users that are in violation of their general terms of use prohibiting illegal activity.

In addition, social media sites should partner with public health agencies and law enforcement by providing Internet surveillance data on suspect online drug sellers and patient safety events. Social media platforms can also follow recommendations of established organizations, such as the National Association of Boards of Pharmacy (NABP) Verified Internet Pharmacy Practice Sites (VIPPS) accreditation program, which accredits online pharmacies that meet pharmacy compliance and licensure requirements in the United States, provides consumer education on safe online drug purchasing, and is the only accreditation program recommended by the FDA [5,10]. This could also include social media surveillance reporting for possible inclusion on the NAPB Not Recommended Sites list that identifies risky online drug sources [10].

Complementing public protection activities, social media sites should not be immune to potential prosecutorial action associated with enabling criminal activity and failing to police obvious false, fraudulent, and illicit marketing that can directly harm patients and consumers. This type of enforcement is consistent with a US $500 million penalty levied by the US Department of Justice against Google regarding allegations of profits from AdWords associated with illegal online drug sales [35]. Large social media companies, such as Facebook, should be legally required to use a portion of their resources for simple public safety enforcement of rules already extant in their policies. In this sense, social media platforms should recognize the goal of promoting public health is consistent with ensuring users are provided a safe online experience.

### Limitations

Our study has certain limitations. We note that data collected from commercially available and convenient Internet services could not be externally validated. However, reliance upon IP address sources would reasonably be considered reliable, especially from known service providers, although IP blocking and redirecting software may be utilized by users and could affect results. Also, we were unable to ascertain why our Google+ study site was suspended. Google+ was a relatively new social media platform at the time of this study; hence, other explanations may be responsible for access issues rather than illicit content, particularly in the context of other clearly legal Google+ accounts disabled without reason. We also did not discern website referral patterns or visitor types of our social media sites, and user traffic could encompass human users and forms of Web technologies (eg, Web bots, crawlers, and indexing services). However, even if all the user traffic generated by our eDTCA sites did not consist of human users, the results still demonstrate our content is being accessed, crawled, indexed, and mapped for use on the Internet. Finally, user traffic was not high volume compared with other e-commerce sites; hence, limiting the generalizability of the study results. Yet our limited costs, knowledge, and mild advertising content still generated global visits.

### Conclusions

The dynamic nature of the Internet and particularly social media results in tremendous challenges for future policy. As the presence, popularity, and diversity of social media continues to expand, social media use by illicit online drug sellers will continue to grow. In response, there will be a need to expand global law enforcement efforts to keep pace with and anticipate suspect online drug seller activities.

As expansion of illegal marketing of prescription drugs online moves from search engines to social media and combination forms [12], it is apparent that these illegal actors are cognizant and evolving with technology. This study highlights the ease of entry into this illicit market and lack of adequate, effective regulation and enforcement. This is an urgent public health concern because legal efforts are continuing to fall behind criminal actions that are nimble and sophisticated. Continued study of the marketing pathways and associated vulnerabilities of digital platforms, such as social media, will be necessary to keep pace with increasing criminal use of the Internet to illegally sell drugs worldwide. Global governance and cooperation is essential between law enforcement, health care stakeholders, and Internet participating parties to address the exploitation of the online sphere by criminals undermining global health.

## Multimedia Appendix 1

Additional information on website terms and conditions and study results.

## Abbreviations

DTCA: direct-to-consumer advertising
eDTCA: online direct-to-consumer advertising
eDTCA2.0: social media–based online direct-to-consumer advertising
FDA: Food and Drug Administration
ICANN: Internet Corporation for Assigned Names and Numbers
IP: Internet protocol
NABP: National Association of Boards of Pharmacy
NPSF: National Patient Safety Foundation
PSM: Partnership for Safe Medicines
VIPPS: Verified Internet Pharmacy Practice Sites
