# Supplementary material for: Global Reach of Direct-to-Consumer Advertising Using Social Media for Illicit Online Drug Sales
Source: J Med Internet Res. 2013 May 29;15(5):e105. doi: 10.2196/jmir.2610 (PMC3668613; doi:10.2196/jmir.2610)
Supplement: Supplementary file 1 [file jmir_v15i5e105_app1.pdf]

Supplementary File

Appendix A: Discussion of Terms and Conditions of eDTCA Sites

General Findings: Beyond low infrastructure costs, our illicit eDTCA Ads remained available despite Internet Service Provider ("ISP") and social media platform terms and conditions generally prohibiting "illegal activities", indicating a lack of surveillance and resources are being utilized by ISPs and social media providers for enforcement against Illicit eDTCA Ads that may violate site requirements.

Facebook T&Cs: Facebook's August 23, 2011 Advertising Guidelines for its fee-based advertisements also has specific guidelines indicating advertisements may not promote prescription drug sales, and online pharmacy advertisements are prohibited except for "certified pharmacies" subject to prior approval by Facebook. Yet our Facebook Ad was clearly promoting the sale of prescription drugs, and a search of Facebook during the study period was not able to ascertain the definition or requirements for a "certified" online pharmacy. This further indicates lax if any enforcement against illegal activities and potential confusion of users regarding site requirements.

Appendix B: Additional Study Results

Additional Facebook Platform Findings: During the study period, our Facebook eDTCA Site's messaging feature allowed an unsolicited Facebook user to contact us requesting further communication. We did not respond to this request, but note that this feature could allow the platform to potentially be used beyond promotion to active communication with other users regarding potential illegal activity.

Geographic Findings: We believe geographic findings of user traffic for this study reflects the wide scope and diverse actors in global illicit drug interactions facilitated by these social media sites. However, although a larger and more focused study (e.g., an actual site selling product) and reliable data on the global prevalence of this criminal activity is necessary to validate this hypothesis. However, presence of lower middle income country traffic for our suspect Ads and Sites is potentially worrisome given rapid growth of Internet use in developing countries, doubling between 2007-2011. These countries may also suffer from lack of robust national public health regulatory systems to warn, detect, and intercept social media-based illicit drug sales.
